# Supplementary material for: Unpacking how teacher support enhances learning engagement in physical education: A serial mediation model of academic self-efficacy and task orientation among Chinese college students
Source: PLoS One. 2025 Oct 6;20(10):e0333813. doi: 10.1371/journal.pone.0333813 (PMC12500086; doi:10.1371/journal.pone.0333813)
Supplement: Attachment — Submitted filename: Response to Reviewers.docx [file pone.0333813.s002.docx]

**Rebuttal letter**

**Manuscript ID:** [PONE-D-25-38320] **Title:** [Unpacking how teacher support enhances learning engagement in physical education: a serial mediation model of academic self-efficacy and task orientation among Chinese college students]

Dear Editor,

We thank you and the reviewers for the insightful comments and constructive suggestions, which have greatly helped us improve our manuscript. We have carefully considered each point and made the corresponding revisions in the manuscript. Below, we provide a point-by-point response to each comment. The specific changes are also available in the “Revised manuscript (tracked changes copy)” file.

**First part, Response Journal Requirements:**

**1.Please ensure that your manuscript meets PLOS ONE's style requirements, including those for file naming. The PLOS ONE style templates can be found at**

**Response:** We sincerely thank the Editor for reminding us to carefully check the manuscript against the PLOS ONE style requirements. We have revised the manuscript text, tables, and figures according to the formatting guidelines, and ensured compliance with the journal’s file naming conventions.

Regarding the in-text citation style, our manuscript currently uses parentheses “( )” instead of brackets “[ ]”. This formatting arises from the citation style settings in our reference management software (EndNote), which cannot be fully converted into bracket style in our local environment. We kindly request the editorial office to assist in converting the parentheses into brackets at the production stage. If required, we are willing to provide the complete reference library file (EndNote) to facilitate this process.

We hope this explanation is acceptable, and we confirm that apart from this point, all other aspects of the manuscript formatting now conform to PLOS ONE requirements.

**2/3. We note that the grant information you provided in the ‘Funding Information’ and ‘Financial Disclosure’ sections do not match. When you resubmit, please ensure that you provide the correct grant numbers for the awards you received for your study in the ‘Funding Information’ section. Please include this amended Role of Funder statement in your cover letter; we will change the online submission form on your behalf.**

**Response:** We thank the Editor for pointing out the inconsistency between the Funding Information and Financial Disclosure sections. We have carefully revised these sections to ensure that the grant numbers and funding details are now consistent. In addition, we have removed the conflicting statement from the manuscript and clarified the corrected funding information in the cover letter.

**4. When completing the data availability statement of the submission form, you indicated that you will make your data available on acceptance. We strongly recommend all authors decide on a data sharing plan before acceptance, as the process can be lengthy and hold up publication timelines. Please note that, though access restrictions are acceptable now, your entire data will need to be made freely accessible if your manuscript is accepted for publication. This policy applies to all data except where public deposition would breach compliance with the protocol approved by your research ethics board. If you are unable to adhere to our open data policy, please kindly revise your statement to explain your reasoning and we will seek the editor's input on an exemption. Please be assured that, once you have provided your new statement, the assessment of your exemption will not hold up the peer review process.**

**Response:** We would like to clarify the Data Availability Statement. The datasets are already publicly accessible in the Figshare repository (DOI: 10.6084/m9.figshare.30084220), as stated in the manuscript. The earlier discrepancy arose because we mistakenly selected the “available upon acceptance” option in the submission system. This has been corrected in the resubmission, and the information is now consistent across both the manuscript and the submission form.

**5. Your ethics statement should only appear in the Methods section of your manuscript. If your ethics statement is written in any section besides the Methods, please delete it from any other section.**

**Response:** We confirm that the ethics statement has been retained only in the Methods section. Any duplicate statements appearing elsewhere in the manuscript have been removed to ensure consistency with the journal’s requirements.

**6. If the reviewer comments include a recommendation to cite specific previously published works, please review and evaluate these publications to determine whether they are relevant and should be cited. There is no requirement to cite these works unless the editor has indicated otherwise.**

**Response:** Neither Reviewer #1 nor Reviewer #2 requested citation of any specific published works. We have carefully re-checked their comments and confirmed that no additional references were suggested. Therefore, no changes were made to the reference list in this regard.

**7. Please review your reference list to ensure that it is complete and correct. If you have cited papers that have been retracted, please include the rationale for doing so in the manuscript text, or remove these references and replace them with relevant current references. Any changes to the reference list should be mentioned in the rebuttal letter that accompanies your revised manuscript. If you need to cite a retracted article, indicate the article’s retracted status in the References list and also include a citation and full reference for the retraction notice.**

**Response:** We have carefully reviewed our entire reference list and confirm that none of the cited papers have been retracted. Therefore, no changes were required in this regard.

**The second part: Response Reviewer's Questions**

**Response to Reviewer 1:**

We sincerely thank Reviewer #1 for the positive evaluation of our study, including the acknowledgment that the quantitative analysis is well conducted and the data provided are sufficient.

**Response to Reviewer 2:**

We greatly appreciate Reviewer #2’s thoughtful comments regarding the theoretical discussion. We agree that the serial mediation mechanism could benefit from deeper theoretical unpacking. Our study was primarily designed as a cross-sectional correlational investigation. Given the broad scope and large, diverse sample, it was not feasible to implement causal comparisons or longitudinal control. Therefore, the focus of this manuscript is to examine the associations among teacher support, academic self-efficacy, sport task orientation, and physical education learning engagement rather than to establish causal effects.

Nevertheless, we found the reviewer’s suggestion very valuable. In response, we have revised the Discussion section to provide stronger theoretical grounding and explanatory depth.

A: added clarifying sentences in the Discussion section to strengthen the explanation of the mechanism and highlight the local educational context;

B: clarified in the rebuttal that the main focus of this paper is to demonstrate the existence and structure of the mechanism rather than to develop an extended theoretical model.

Specifically, we analyzed the mechanism from two theoretical perspectives—Self-Determination Theory (SDT) and Social Cognitive Theory (SCT) combined with Achievement Motivation Theory (AMT)—to clarify why teacher support enhances academic self-efficacy and task orientation, which in turn foster learning engagement. These additions strengthen the theoretical basis of our model and enhance the explanatory power of the findings while remaining consistent with the scope of the study.

**The specific revision process is as follows**：

1. line 419-422 (Track Changes): add, This suggests that teacher support first strengthens students’ academic confidence, and this confidence motivates them to adopt a mastery-focused task orientation. Such task orientation channels self-belief into sustained effort, ultimately leading to greater PELE.

**Statement:** In the Interpretation of findings, add the logical chain explaining why ASE → STO → PELE.

2. line 456-460 (Track Changes): Together, these perspectives explain the sequential pathway: SDT highlights that teacher support satisfies basic needs and builds ASE; SCT emphasizes that ASE enhances students’ motivation to persist; AMT clarifies how STO transforms this confidence into mastery-driven engagement.

**Statement:** In the Theoretical implications, add a linked explanation of the three theories rather than listing them separately.

3. line 487-491 (Track Changes): In Chinese universities, PE is often exam-oriented and teacher-centered, which means students rely heavily on teacher encouragement to build ASE. Once this confidence is established, it becomes a key driver for adopting STO, as students gradually shift from compliance to active mastery.

**Statement:** In the Critical analysis, add the unique features of the TS → ASE → STO pathway in the Chinese context.
